# Bioinformatics analysis combined with untargeted metabolomics reveals lipid metabolism-related genes and their biological markers in chronic spontaneous urticaria

**DOI:** 10.3389/fgene.2025.1550205

**Published:** 2025-08-18

**Authors:** Zhiming Hu, Qiong Wang, Yuqi Wang, Yao Gao, Jianhua Hao, Rui Li, Hua Zhao, Shuping Guo, Hongzhou Cui

**Affiliations:** ^1^ Department of Dermatology, First Hospital of Shanxi Medical University, Taiyuan, China; ^2^ The First Clinical Medical College of Shanxi Medical University, Taiyuan, China; ^3^ Dermatology Department, Changzhi Second People’s Hospital, Changzhi, China

**Keywords:** bioinformatics analysis, chronic spontaneous urticaria, lipid metabolism, untargeted metabolomics, arachidonic acid, immunity, biomarkers

## Abstract

**Background:**

Chronic spontaneous urticaria (CSU) is an immune-driven skin condition with a multifaceted and not yet fully understood pathogenesis. Although substantial research has been conducted, viable therapeutic targets are still scarce. Studies indicate that disruptions in lipid metabolism significantly influence the development of immune-related disorders. Nevertheless, the precise relationship between lipid metabolism and CSU remains underexplored, warranting further investigation.

**Methods:**

We obtained the GSE72540 and GSE57178 datasets from the Gene Expression Omnibus (GEO) repository. For the GSE72540 dataset, we identified differentially expressed genes (DEGs) and performed weighted gene co-expression network analysis (WGCNA) on them. The identified DEGs were cross-referenced with lipid metabolism-related genes (LMRGs). To identify hub genes, we constructed a protein-protein interaction (PPI) network. These hub genes were validated using the GSE57178 dataset to identify potential diagnostic markers. Additionally, gene set enrichment analysis (GSEA) and receiver operating characteristic (ROC) curve analysis were employed to evaluate their diagnostic potential. In the CSU mouse model, we further validated the expression levels of these hub genes. Finally, untargeted metabolomics was conducted to detect lipid metabolism-related metabolites in the serum of CSU patients.

**Result:**

Using bioinformatics analysis, three hub genes were identified: *SLC2A4*, *PTGS2*, and *PLA2G2A*. In skin tissues from CSU-like mouse models, the mRNA levels of *PTGS2* and *PLA2G2A* were significantly upregulated compared to the control group. Additionally, untargeted metabolomics revealed 60 distinct lipid metabolites, with a marked increase in arachidonic acid levels observed in the CSU group.

**Conclusion:**

*PTGS2* and *PLA2G2A* are key hub genes for CSU, and arachidonic acid can serve as a potential serum biomarker.

## 1 Introduction

Chronic spontaneous urticaria (CSU) is a prevalent and distressing immune-related skin condition, marked by recurrent episodes of intense itchy wheals and angioedema (Zuberbier et al., 2018). Affecting around 1% of the global population, CSU’s prevalence continues to grow (Fricke et al., 2020). The condition severely impacts patients’ quality of life and presents treatment challenges, contributing to a considerable economic burden (Kolkhir et al., 2022b). Many individuals endure a prolonged disease course, often spanning several years, with nearly 20% experiencing recurrent flare-ups separated by symptom-free intervals (Curto-Barredo et al., 2018). Current therapeutic options, including antihistamines or biologics, are limited by potential side effects such as sedation and the need for high doses, with some patients remaining treatment-refractory to these treatments (Greiner et al., 2022; Maurer et al., 2022). These challenges underscore the need to explore new therapeutic targets to improve CSU management.

In 2004, Han et al. formally defined “hub” proteins within yeast PPI networks as highly connected nodes in scale-free networks (Han et al., 2004). The specific term “hub gene” was subsequently introduced in 2006, referring to genes exhibiting high connectivity in weighted co-expression networks that are essential for yeast viability (Carlson et al., 2006), later definitions broadened this concept to genes engaging in extensive interactions within gene networks (Yu et al., 2017). With the continuous advancement of bioinformatics, hub genes have gained increasing research relevance due to their critical roles in gene regulation and biological processes (Cooper et al., 2006; Yu et al., 2017). For instance, transcription factors (TFs)—proteins that bind specific DNA sequences to regulate target genes—often function as hub genes. Approximately 10% of human genes encode 2,600 TFs (Wei and Pan, 2008), which collectively drive the majority of genomic regulatory activities, particularly during development. Consequently, genes encoding TFs (TF-coding genes) are typically regarded as hub genes. In cancer research, oncogenes and tumor suppressor genes involved in tumorigenesis may act as hub genes within tumor genetic networks (Li and Xie, 2015; Selvanathan et al., 2015). Similarly, *TRIB3* has been identified as a hub gene exacerbating steatohepatitis, lipid accumulation, and inflammation *in vitro* (Xia et al., 2025). While ten hub genes *(SFRP4, FMOD, HAPLN1, LTBP2, SVEP1, BCL6, ANPEP, CD38, ATRNL1* and *BEX1*) correlate with the co-occurrence of atrial fibrillation (AF) and heart failure (HF) (Zhou et al., 2025). Consequently, the identification of hub genes serves as an effective strategy for elucidating core regulatory mechanisms in complex biological processes, discovering potential key therapeutic targets, and developing diagnostic biomarkers, thereby explaining the broad biological significance and substantial research value driving sustained scientific interest in hub gene investigations.

CSU is a complex disease centered on aberrant mast cell activation, involving multifactorial interactions such as immune dysregulation, autoantibodies, and environmental triggers. Its pathophysiological process fundamentally constitutes a highly interconnected network encompassing immune cell activation, inflammatory mediator release, autoantibody generation, complement and coagulation system activation, and metabolic alterations (Kaplan et al., 2023; Oliver and Saini, 2024). Although second-generation antihistamines are first-line treatments for CSU, nearly 50% of patients show inadequate symptom control even with standard or quadrupled doses (Kaplan et al., 2023). Targeting hub genes (e.g., *PTGS-2, IL-6*) may yield novel diagnostic and therapeutic approaches (Chen et al., 2024; Su et al., 2023), as these genes typically occupy critical nodes in regulatory networks. Modulating them could effectively restore dysregulated network states.

Lipids, consisting of fats and oils, act as secondary messengers in intracellular signaling and are essential for the proper functioning of various organelles (Olzmann and Carvalho, 2019). Lipid metabolism encompasses the processes of lipid digestion, absorption, catabolism, and utilization, involving key molecules such as triglycerides (TG), cholesterol, and fatty acids (FAs) (Salciccia et al., 2021). Additionally, lipids serve as precursors for bioactive molecules with crucial biological functions. For instance, polyunsaturated fatty acids (PUFAs) undergo enzymatic oxidation to generate lipid mediators. Arachidonic acid (AA), an omega-6 PUFA, gives rise to pro-inflammatory derivatives like prostaglandins (PGs), thromboxanes, and leukotrienes (Ye et al., 2021). These lipid mediators play a key role in the pathogenesis of allergic diseases such as asthma, allergic rhinitis, urticaria and food allergies (Schauberger et al., 2016). Both lipid mediators and histamine, which are released by mast cells and basophils, drive the inflammatory processes involved in CSU (Kabashima et al., 2018). While lipid metabolism-related genes (LMRGs) have been associated with allergic diseases such as asthma and allergic rhinitis (Ding et al., 2023; Tao et al., 2023), the specific association between LMRGs and CSU remains largely unexplored.

Metabolomics is an emerging discipline in today’s life sciences that utilizes advanced analytical chemistry tools and complex statistical approaches to profile small-molecule metabolites (<1500 Da) present within specific cells, tissues, or organisms (Huang et al., 2022). These metabolites are essential to cellular processes, contributing to enzyme-driven chemical reactions and supporting various cellular functions (Schrimpe-Rutledge et al., 2016). In pathological conditions, cellular metabolism becomes dysregulated, triggering enhanced catabolic activity, suppressed anabolic pathways, and the release of pro-inflammatory mediators (Tuerxun et al., 2024). As a result, metabolomics holds promise for uncovering biomarkers and elucidating biological pathways involved in CSU.

In this study, we obtained datasets related to CSU from the Gene Expression Omnibus (GEO) database to perform bioinformatics analysis, focusing on LMRGs linked to CSU. Hub genes were identified, and a CSU mouse model was developed to verify the differential expression of these genes compared to control groups. Furthermore, we conducted untargeted metabolomics to examine lipid metabolism-related metabolites in serum samples from CSU patients, adding metabolic insights to our findings. By integrating bioinformatics with metabolomics data, we identified potential biomarkers that could contribute to the understanding and diagnosis of CSU.

## 2 Materials and methods

### 2.1 Processing of data

We accessed the Gene Expression Omnibus (GEO) database, managed by the National Center for Biotechnology Information (NCBI) (http://www.ncbi.nlm.nih.gov/geo, accessed on February 1, 2024), which houses extensive gene expression profiles and high-throughput data from various experimental studies. Through systematic searches using keywords like “urticaria” and “chronic urticaria,” we identified two relevant datasets: GSE57178 and GSE72540. The GSE57178 dataset contains mRNA expression data from lesional skin samples of CSU patients (N = 6) and healthy controls (N = 5). The GSE72540 dataset includes data from lesional skin of CSU patients (N = 10) and healthy controls (N = 8). All CSU patients included in these datasets exhibited active disease. We utilized the GSE72540 dataset as the internal training set and the GSE57178 dataset as the external validation set for our analysis.

The raw microarray gene expression data were dealt with using the “Affy” package and the Robust Multi-array Average (RMA) algorithm. Probes were mapped to gene symbols according to platform-specific annotation files, with those lacking corresponding gene symbols excluded from further analysis. For genes targeted by multiple probes, the mean expression value was calculated and used for downstream analysis. Additionally, we obtained 829 LMRGs from the Molecular Signatures Database (MSigDB) (https://www.gsea-msigdb.org/gsea/msigdb, accessed on 15 February 2024).

### 2.2 Recognition of differentially expressed genes (DEGs)

In our study, we utilized the “limma” package (version 3.40.6) to perform differential expression analysis, identifying DEGs between skin samples of CSU and healthy. Genes with a fold change (FC) > 1.5 and an adjusted *p*-value <0.05 were statistically significant and selected as DEGs for further analysis.

### 2.3 Recognition of CSU-Related genes (CSU-RGs)

We applied weighted gene co-expression network analysis (WGCNA) to the GSE72540 dataset to identify CSU-related genes (CSU-RGs) (Langfelder and Horvath, 2008). First, sample clustering was performed to detect and troubleshooting anomalous samples. Next, we determined the optimal soft-thresholding power (β). Using the selected β, gene modules were identified through dynamic tree cutting. The correlation between these modules and CSU was evaluated, and for clinically relevant modules, we calculated and visualized both gene significance (GS) and module membership (MM). After screening, the module with the most significant correlation with CSU phenotype was identified, which was identified as a key module and selected for deep study.

### 2.4 Identification and functional enrichment analysis of CSU-Lipid metabolism related DEGs (CSU-LM-DEGs)

CSU-LM-DEGs were identified by intersecting LMRGs, key module genes from the WGCNA analysis, and the DEGs from the GSE72540 dataset. Enrichment analysis of the CSU-LM-DEGs was subsequently conducted using the Metascape database (https://metascape.org/gp/index.html, accessed on 03 April 2024), with a significance threshold of *P <* 0.05 (Wu et al., 2021).

### 2.5 Construction of the PPI (protein-protein interaction network) of CSU-LM-DEGs and screening of hub genes

The PPI network for CSU-LM-DEGs was constructed using the Search Tool for the Retrieval of Interacting Genes (STRING) database (https://string-db.org/, accessed on 13 April 2024), confidence score threshold >0.4. The resulting network was then visualized using Cytoscape to facilitate further analysis and interpretation. Hub genes are operationally defined as the top 10 of genes ranked by connectivity (i.e., the number of directly interacting genes/proteins) in a PPI network, where nodes represent genes and edges denote functional associations. The ranking was based on multiple centrality measures, including Maximal Clique Centrality (MCC), Degree, Stress, and Edge Percolated Component (EPC), ensuring a comprehensive selection of key genes within the PPI network.

### 2.6 Immune analysis

We used the CIBERSORT algorithm (https://cibersortx.stanford.edu/, accessed on 15 April 2024) to investigate in detail the differences in immune cell infiltration between CSU patients and healthy controls in the training set. Using the Wilcoxon test, we identified immune cell types that showed significant differences between CSU patients and healthy controls. Additionally, we employed the Spearman rank correlation coefficient to calculate the correlations between hub genes and immune cells, identifying immune cells significantly associated with hub genes.

### 2.7 Validation in GEO datasets

We validated the expression value of potentially hub genes in the GSE57178 dataset using the Wilcoxon test, with a significance threshold of *p*-value <0.05. This analysis enabled the identification and selection of hub genes based on their expression between CSU patients and healthy controls.

### 2.8 Gene set enrichment analysis (GSEA) and receiver operating characteristic (ROC) curve analysis of hub genes

GSEA was performed using annotated hallmark gene sets on the SangerBox platform (http://sangerbox.com/, accessed on 23 April 2024), to explore the biological functions associated with hub genes in CSU (Powers et al., 2018). A *p*-value <0.05 was set as the threshold for statistical significance. To evaluate the diagnostic reliability of these genes, ROC curves were made, with an area under the curve (AUC) ≥ 0.85 indicating strong diagnostic potential.

### 2.9 Validation in the CSU-like mouse model

#### 2.9.1 Animals and grouping

We purchased 6-week-old male BALB/c mice from GemPharmatech Ltd., which had free access to tap water and rodent food, were placed in a controlled environment (22°C–24°C) with alternating light and darkness for 12 h. After a 1-week adaptation period, the mice were randomly divided into the normal control group and the experimental group (n = 8 per group). In the control group mice, 0.1 mL saline was intraperitoneal (ip) injection, while the model group was administered 0.1 mL saline containing 0.1 mg ovalbumin (OVA) and 100 mg aluminum hydroxide adjuvant on day 1, with a second identical injection on day 21 (Sun et al., 2023). On day 27, all mice were anesthetized with sodium pentobarbital (60 mg/kg, ip) for sample collection. Afterward, humane euthanasia was carried out via cervical dislocation following sample extraction. Experiments were conducted in the Animal Experiment Center of Shanxi Medical University. Experiments were approved by the Institutional Animal Care and Use the Animal Management Committee of the First Hospital of Shanxi Medical University (NO: DWLL-2024-033) and conducted in accordance with the National Institutes of Health Guidelines for the Care and Use of Laboratory Animals and with the ARRIVE guidelines. Efforts were made to reduce the number of animals used and to minimize animal pain and discomfort.

#### 2.9.2 Scratching behavior analyses

The severity of itching (including scratching latency, duration, and frequency) was monitored within 30 min following the second immunization in all groups. Signs of itching included head scratching with the front paws, scratching of the trunk post-immunization, and biting of various body parts (Choi et al., 2018).

#### 2.9.3 Enzyme-linked immunosorbent assay (ELISA)

The collected blood samples were centrifuged at 4,000 rpm for 10 min to separate the serum. Serum immunoglobulin E (IgE) levels were quantified using ELISA kits (JianChen Bioengineering Institute, Nanjing, China). The optical densities were measured at 450 nm using a microplate reader (Thermo Fisher, Waltham, MA, United States), and IgE concentrations were calculated based on the standard curve.

#### 2.9.4 Histopathological analysis

For histopathological analysis, dorsal skin lesions were excised, fixed in 10% paraformaldehyde for 48 h, dehydrated using ethanol, and embedded in paraffin wax. Tissue sections of 3 μm thickness were prepared and treated with xylene and ethanol, followed by staining with hematoxylin and eosin (H&E) (Wuhan Servicebio Technology Co., Ltd., Wuhan, China) to evaluate edema and inflammatory cell infiltrat ion. Images were randomly captured by a pathologist using light microscopy (Nikon Eclipse E100, Tokyo, Japan) at ×100 magnification (×10 ocular and ×10 objective lenses). The degree of edema, telangiectasia, and inflammatory cell infiltration was assessed using a four-point grading system. The degree of edema, telangiectasia, and inflammatory cell infiltration was scored on a subjective scale of 0–3 as follows which from three fields per hematoxylin and eosin-stained sections: 0, no edema; no telangiectasia; no inflammatory cell infiltration; 1, slight edema; slight telangiectasia; slight inflammatory cell infiltration; 2, moderate edema; moderate telangiectasia; moderate inflammatory cell infiltration; 3, severe edema; severe telangiectasia; severe inflammatory cell infiltration. The histologic score of each animal was the average of the total scores of 3 pathological features from 3 visual fields.

For mast cell detection, skin tissue samples were fixed in 10% paraformaldehyde, dehydrated through a graded ethanol series, embedded in paraffin, and sectioned. Sections were stained with toluidine blue solution for 10 min at 37°C and subsequently mounted with resin. Mast cells within the stained sections were quantified by counting in three randomly selected fields of view per section under a light microscope (Peng et al., 2021).

#### 2.9.5 Real-time quantitative polymerase chain reaction (RT-qPCR)

Our kits were purchased from Beijing TransGen Biotech Ltd. According to the instructions of the kits, firstly, total RNA was extracted from mouse skin tissues using a total RNA extraction kit, and then reverse transcribed into cDNA using a reverse transcription kit, and then analyzed by quantitative PCR (qPCR) on the Bio-Rad real-time PCR Detection System (Hercules, CA, United States) using PerfectStart Green qPCR SuperMix. Primer sequences are detailed in the Supplementary Table S1.

### 2.10 Participant selection and criteria

A total of 35 patients diagnosed with CSU from the Dermatology Department at the First Clinical College of Shanxi Medical University, along with 21 healthy volunteers recruited from the physical examination center, participated in the study. Both groups were matched in terms of age. The inclusion criteria for CSU patients were: (1) Age range of 18–75 years; (2) A CSU diagnosis was established according to EAACI/GA^2^LEN/EuroGuiDerm/APAAACI guidelines (Apaer et al., 2024). Asthma, and Immunology. Participants were excluded if they had used antibiotics, aspirin/NSAIDs, leukotriene receptor antagonists, 5-lipoxygenase inhibitors, systemic steroids, or biologics in the past month, presented abnormal coagulation markers, or had any pre-existing conditions, such as allergic rhinitis, atopic dermatitis, asthma, eczema, obesity, or diabetes. This study received approval from the Ethics Committee of the First Hospital of Shanxi Medical University in compliance with the Declaration of Helsinki under procedure number NO. KYLL-2023-120. Prior to commencement, all participants were provided with a detailed verbal and written explanation of the study and gave informed consent by signing a consent form.

#### 2.10.1 Serum sample processing and non-targeted metabolomics analysis

Following centrifugation of the collected blood samples, the supernatant serum was carefully harvested and preserved at −80°C for subsequent analysis.100 μL of serum sample was added to the EP tube followed by 400 μL of pure methanol for protein precipitation. The sample was then vortexed well and left on ice for 5 min, then centrifuged and the supernatant was collected and diluted with chromatography grade water to bring the methanol content to 60%. The solution was filtered through a 0.22 mm filter membrane, centrifuged again, and finally the filtrate was collected and used for LC-MS analysis. A blank sample was prepared using 60% methanol containing 0.1% formic acid as a control, and the pretreatment steps were the same as the experimental samples.

The raw data files (.raw) generated from UHPLC-MS/MS were analyzed using Compound Discoverer 3.0 (CD 3.0, Thermo Fisher). The identified compounds were cross-validated with the mzCloud database (https://www.mzcloud.org/, accessed May 3, 2024) and the ChemSpider database (http://www.chemspider.com/, accessed May 4, 2024).

### 2.11 Statistical analysis

Statistical analyses were performed using GraphPad Prism 10. When the data were normally distributed and the variance was homogeneous, the comparison between groups was performed using t-test. Non-parametric Mann-Whitney U test was applied when variance was uneven. A *P*-value of <0.05 was considered statistically significant.

## 3 Result

### 3.1 Based on bioinformatics results analysis

#### 3.1.1 Identification of DEGs

**FIGURE 1:**
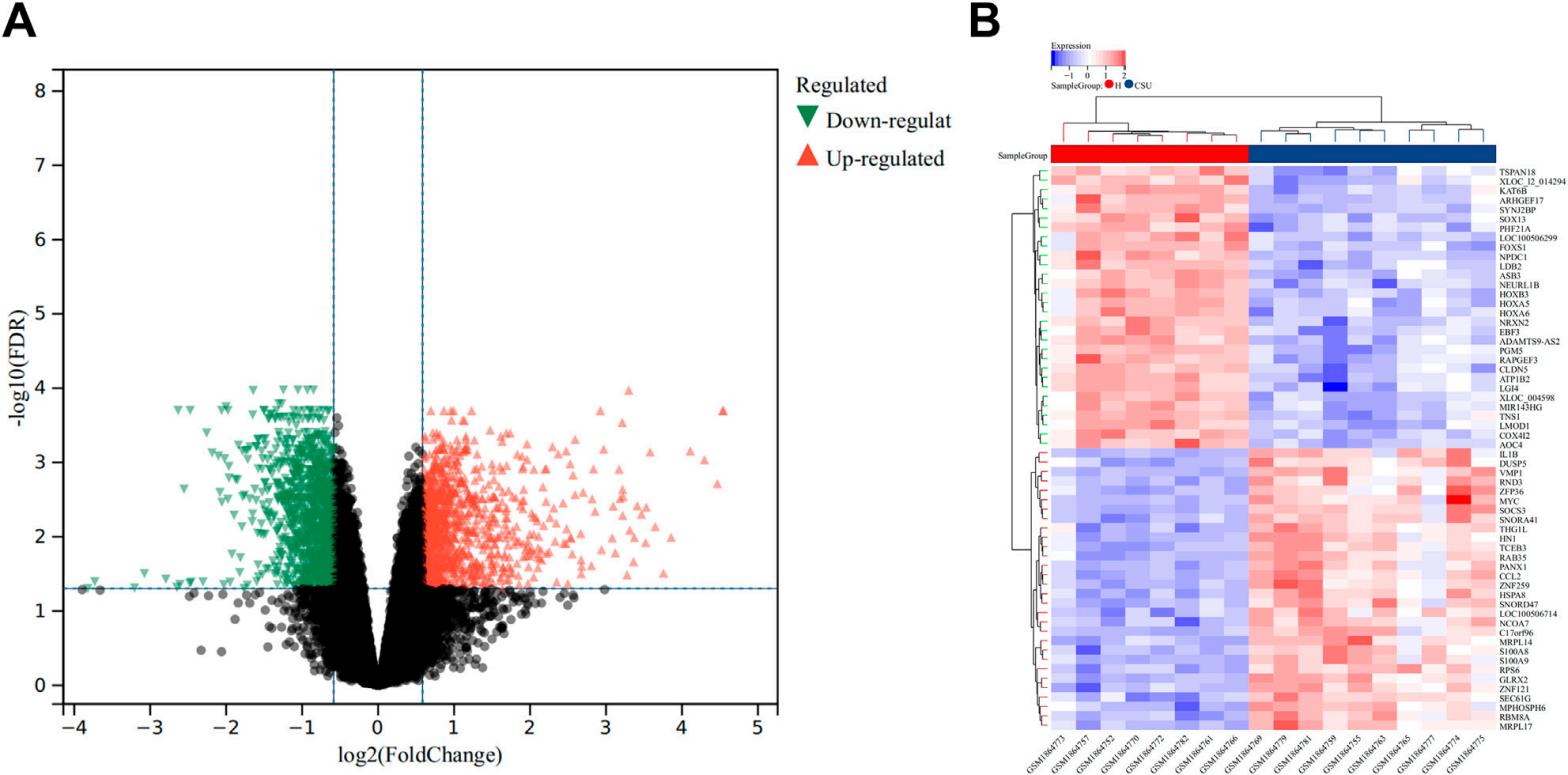
Analysis of Differentially Expressed Genes (DEGs). **(A)** Volcano plot illustrating DEGs between the CSU and healthy (H) control groups. **(B)** Heatmap depicting the top 50 upregulated and downregulated DEGs.

In the GSE72540 dataset, a total of 2309 DEGs were detected between CSU and healthy control samples by setting FDR<0.05 and FC > 1.5 criteria. Specifically, 1,058 genes were upregulated, while 1,251 were downregulated. Visual representations of these findings are provided in Figure 1A, which displays the volcano plot of all 2,309 DEGs, and Figure 1B, which shows a heatmap featuring the top 60 DEGs.

#### 3.1.2 Identification of CSU-RGs

**FIGURE 2:**
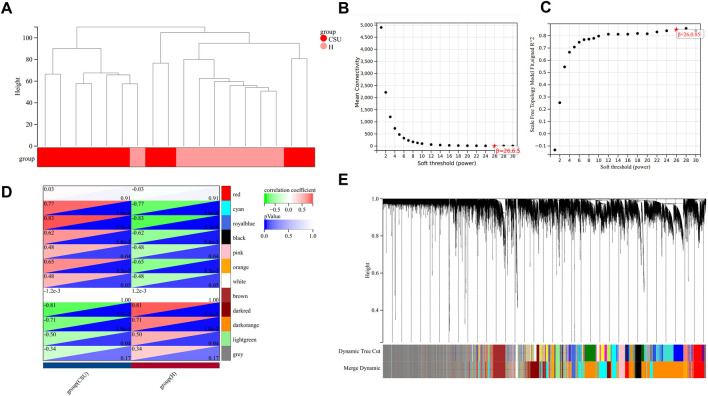
Weighted Gene Co-expression Network Analysis (WGCNA). **(A)** Clustering dendrogram of samples. **(B,C)** Network topology analysis for various soft-thresholding powers. **(D)** Heatmap showing the association between CSU and healthy (H) control groups. **(E)** Clustering dendrogram of genes.

WGCNA was conducted on the GSE72540 dataset to identify CSU-related genes (CSU-RGs), with visualization performed using the Sangerbox platform (http://vip.sangerbox.com/home.html, accessed April 3, 2024). Sample clustering analysis confirmed that no outliers were present (Figure 2A). The soft threshold (β) was set to 26 (Figures 2B,C), e0ach module contains 100 genes. A total of 12 distinct modules were identified, each represented by a unique color (Figures 2D,E). Modules with a correlation coefficient >0.7 and *P* < 0.05 were considered significantly associated with CSU. Four such modules—cyan, royalblue, darkred, and darkorange—were identified (Figure 2D). From these modules, 3,130 CSU-related genes (CSU-RGs) were extracted.

#### 3.1.3 Identification and functional enrichment analysis of CSU-Lipid metabolism related DEGs (CSU-LM-DEGs)

**FIGURE 3:**
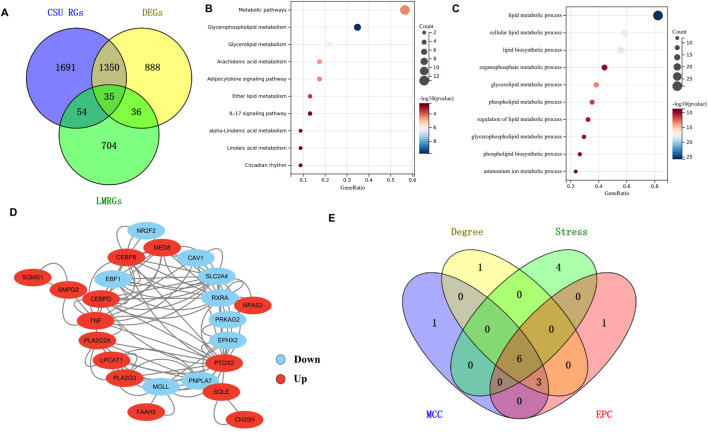
Analysis of CSU-Lipid Metabolism Related DEGs (CSU-LM-DEGs) and Identification of Hub Genes. **(A)** Venn diagram illustrating the intersecting genes among Differentially Expressed Genes (DEGs), CSU-Related Genes (CSU-RGs), and lipid metabolism-related genes (LMRGs). **(B,C)** Bubble charts showing Kyoto Encyclopedia of Genes and Genomes (KEGG) and Gene Ontology (GO) analyses of the 35 intersecting genes. **(D)** The PPI network of 35 CSU-LMR DEGs constructed using the STRING database. **(E)** A Venn diagram displaying the intersecting genes obtained from four different algorithms.

As depicted in the Venn diagram (Figure 3A), the intersection of 3,130 CSU-RGs, 829 LMRGs, and 2,309 DEGs resulted in the identification of 35 CSU-LM DEGs. Kyoto Encyclopedia of Genes and Genomes (KEGG) and Gene Ontology (GO) enrichment analyses (Figures 3B,C) highlighted several key metabolic pathways, including glycerophospholipid metabolism, glycerolipid metabolism, arachidonic acid metabolism, and the adipocytokine signaling pathway. The main biological processes identified were cellular lipid metabolism, lipid biosynthesis, organophosphate metabolism, glycerolipid metabolism, and phospholipid metabolism.

#### 3.1.4 Identification of hub genes

A PPI network was created for the 35 CSU-LM-DEGs, with isolated proteins removed. The final PPI network is displayed in Figure 3D. Among the key nodes, *PTGS2* exhibited interactions with several other proteins, including *PLA2G2A, TNF,* and *CEBPB*. To determine the hub genes, the top 10 genes identified by four distinct algorithms were cross-referenced. This intersection yielded six hub genes (Figure 3E): *CEBPB, PLA2G2A, TNF, SLC2A4, RXRA,* and *PTGS2.*

#### 3.1.5 Immune cell infiltration analysis

**FIGURE 4:**
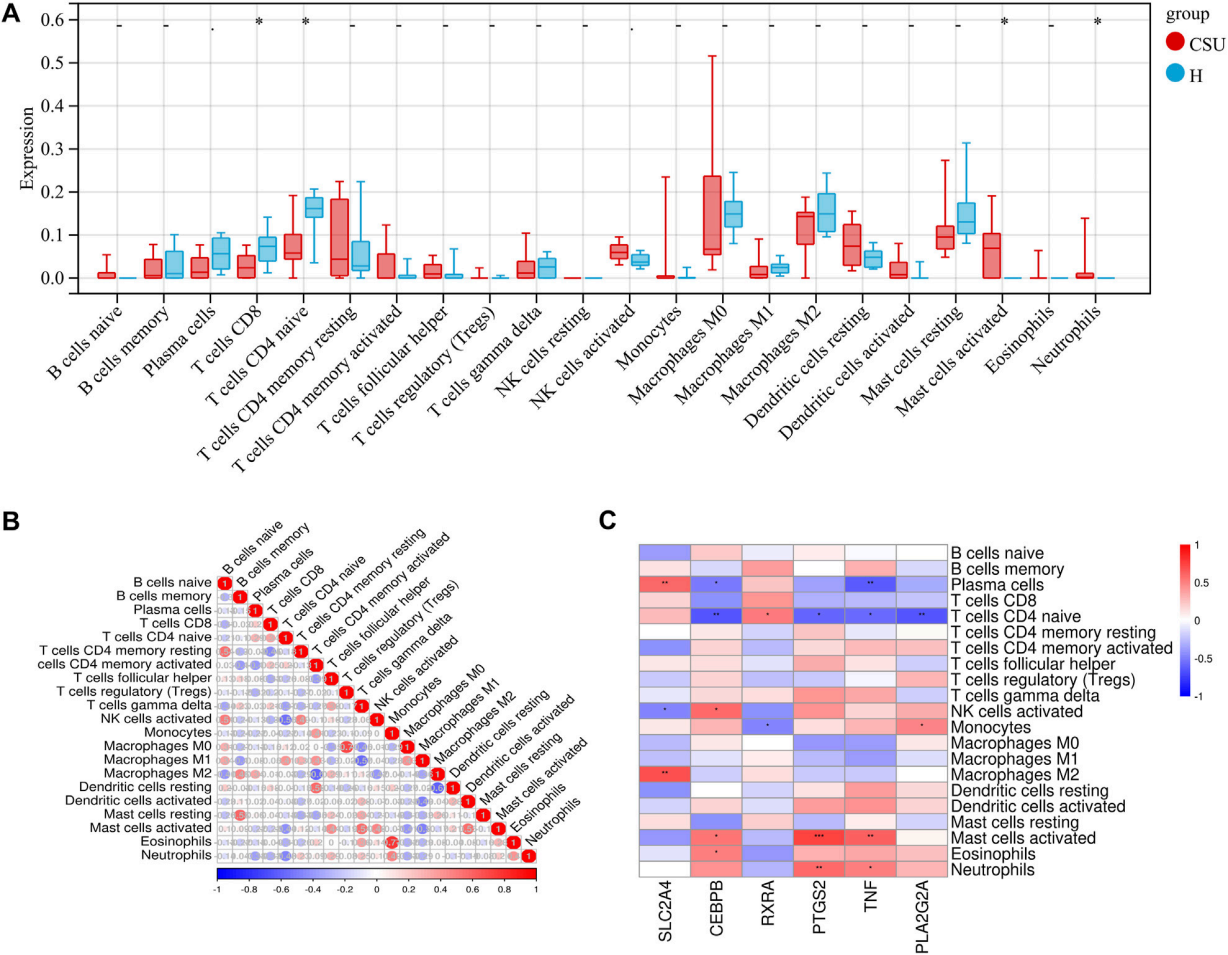
Immuno-infiltration analysis in CSU. **(A)** Differences in immune cell infiltration between the CSU group and the healthy (H) control group. **(B)** Heatmap of immune cell correlationsBlue indicates negative correlations, red indicates positive correlations, and deeper colors represent stronger correlations. **(C)** Heatmap of correlations between hub genes and immune cells (*P < 0.05, **P < 0.01, ***P < 0.001).

CSU is widely recognized as an immune-mediated disease. To investigate differences in immune cell composition between CSU patients and healthy controls, we applied the CIBERSORT algorithm to perform immune cell infiltration analysis. Figure 4A illustrates the distribution of 22 immune cell types in lesional skin samples from CSU patients and healthy controls. Compared to healthy controls, lesional samples from CSU patients showed a significant increase in activated mast cells and neutrophils (*P <* 0.05), along with a reduction in CD8^+^ T lymphocytes and naïve CD4^+^ T lymphocytes (*P <* 0.05). Figure 4B depicts the correlations between different immune cell types in lesional skin samples from CSU patients and healthy controls. Activated mast cells were negatively correlated with CD8^+^ T lymphocytes but positively correlated with activated NK cells and neutrophils.

Next, we conducted an in-depth analysis of the relationship between immune cell infiltration levels and key hub genes in the skin samples, as shown in Figure 4C. Specifically, the expression of *SLC2A4* was positively correlated with the numbers of CD8^+^ T lymphocytes and naïve CD4^+^ T lymphocytes but negatively correlated with activated mast cells. *RXRA* exhibited a similar expression pattern, showing positive correlations with CD8^+^ T lymphocytes and naïve CD4^+^ T lymphocytes but negative correlations with activated mast cells and neutrophils. In contrast, the expression levels of *CEBPB*, *PTGS2*, *TNF*, and *PLA2G2A* were negatively correlated with CD8^+^ T lymphocytes and naïve CD4^+^ T lymphocytes but positively correlated with activated mast cells and neutrophils. Moreover, they reveal a strong association between these hub genes and immune cells. These results offer valuable insights and may open new avenues for future CSU research.

#### 3.1.6 The expression of hub genes in the validation set

**FIGURE 5:**
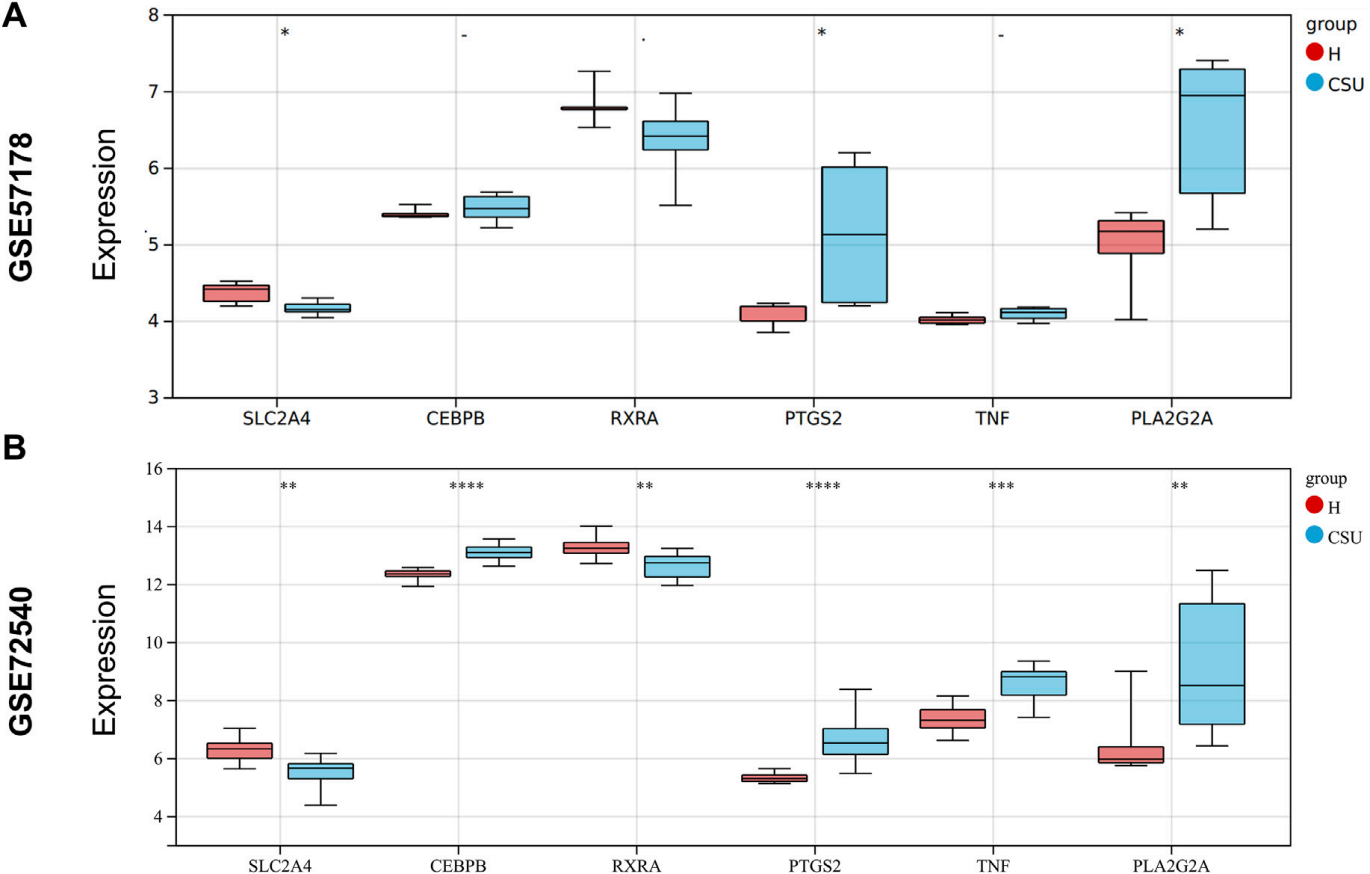
Expression Levels of Hub Genes: **(A)** Expression levels of hub genes in the GSE57178 validation set; **(B)** Expression levels of hub genes in the GSE72540 training set (*P < 0.05, **P < 0.01, ***P < 0.001, ****P < 0.0001).

The expression levels of the hub genes were validated using the GSE57178 validation dataset and the GSE72540 training dataset, as shown in Figures 5A,B. In comparison to the control group, *PTGS2* and *PLA2G2A* were significantly upregulated in the CSU group (*P <* 0.05), while *SLC2A4* was notably downregulated (*P <* 0.05). These results suggest that these three genes may act as lipid-associated hub genes for CSU pathogenesis.

#### 3.1.7 GSEA and ROC curve analysis of hub genes

**FIGURE 6:**
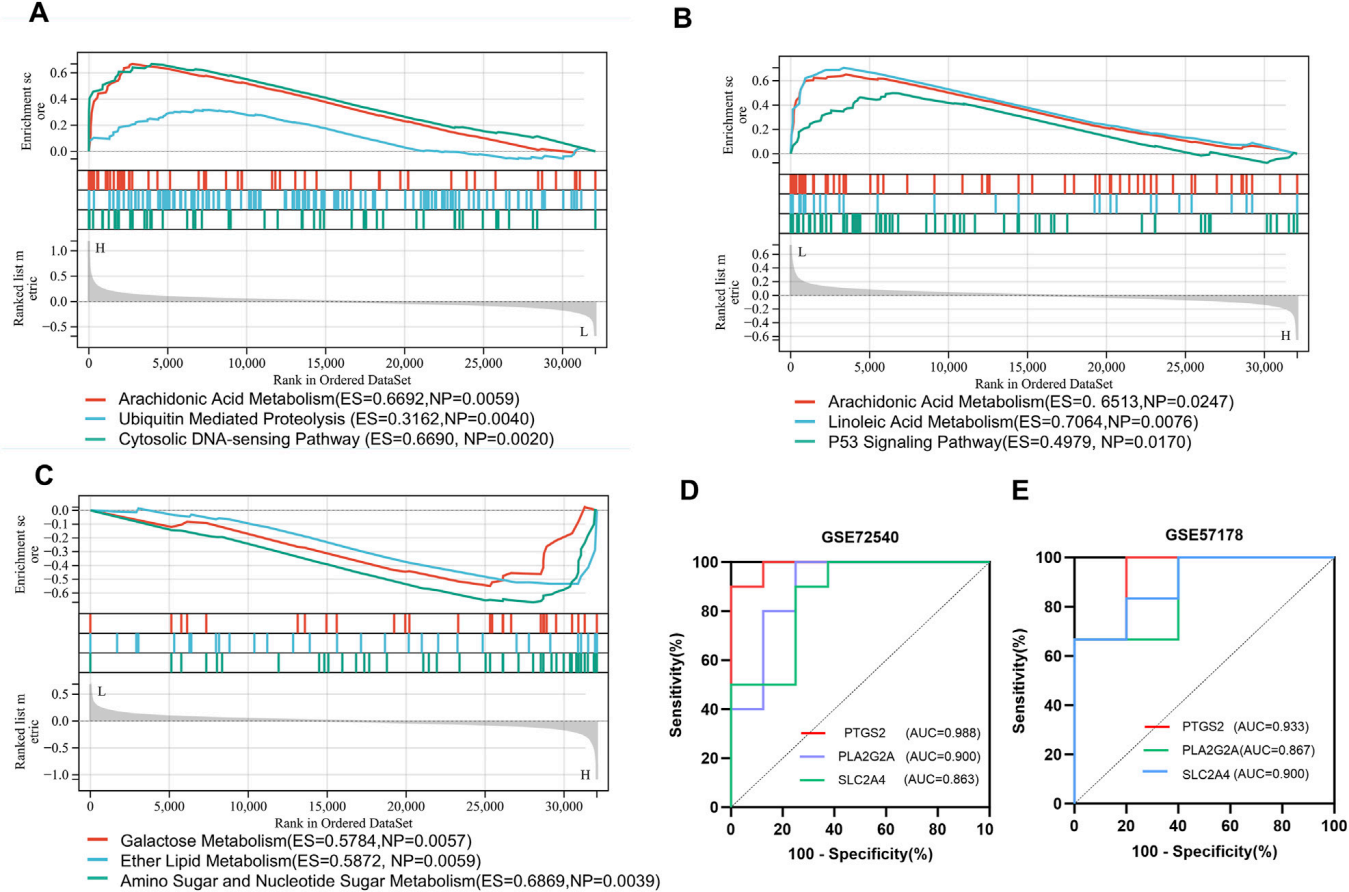
Gene Set Enrichment Analysis (GSEA) and Receiver Operating Characteristic (ROC) Curve Analysis: **(A–C)** Gene Set Enrichment Analysis (GSEA) enrichment plots for genes (*PTGS2*, *PLA2G2A*, *SLC2A4*); **(D,E)** ROC analysis of hub genes (PTGS2, *PLA2G2A*, *SLC2A4*) in the GSE72540 and GSE57178 datasets.

To further explore the influence of hub genes on CSU, we used GSEA to explore relevant signaling pathways. The top three enriched pathways for each gene are displayed in Figures 6A–C. For *PTGS2* (Figure 6A), the enriched pathways include arachidonic acid metabolism, ubiquitin-mediated proteolysis, and the cytosolic DNA-sensing pathway. As shown in Figure 6B, the primary pathways enriched for *PLA2G2A* are arachidonic acid metabolism, linoleic acid metabolism, and the p53 signaling pathway. Meanwhile, the top enriched pathways for *SLC2A4* (Figure 6C) include amino sugar and nucleotide sugar metabolism, ether lipid metabolism, and galactose metabolism. Notably, arachidonic acid metabolism was identified as a shared pathway between *PTGS2* and *PLA2G2A*, highlighting its potential importance in the pathogenesis of CSU.

### 3.2 Based on animal experiment results

#### 3.2.1 Behavioral and pathological changes in skin tissue from an OVA-Induced mouse model

As shown in Supplementary Figure S1A, compared to the healthy control group, OVA-Induced mice exhibited a significantly shortened scratching latency (*P <* 0.0001), along with dramatically increased scratching duration and frequency within 30 min (*P <* 0.0001). Serum IgE levels were also significantly elevated in the OVA-Induced group (*P <* 0.0001) (Supplementary Figure S1B). Histological analysis revealed minimal edema, capillary dilation, or inflammatory cell infiltration in control tissues. In contrast, the OVA-Induced group displayed pronounced edema, capillary dilation, and inflammatory cell infiltration, with significantly higher histopathological scores than the normal group (*P <* 0.0001) (Supplementary Figure S1C). To assess mast cell infiltration, we performed toluidine blue staining. As demonstrated in Supplementary Figure S1D, mast cell numbers were significantly increased in the OVA-Induced group compared to healthy controls (*P* < 0.0001). These results indicate the successful establishment of our CSU-like mouse model.

#### 3.2.2 Validation of hub genes in the CSU-like mouse model

**FIGURE 7:**
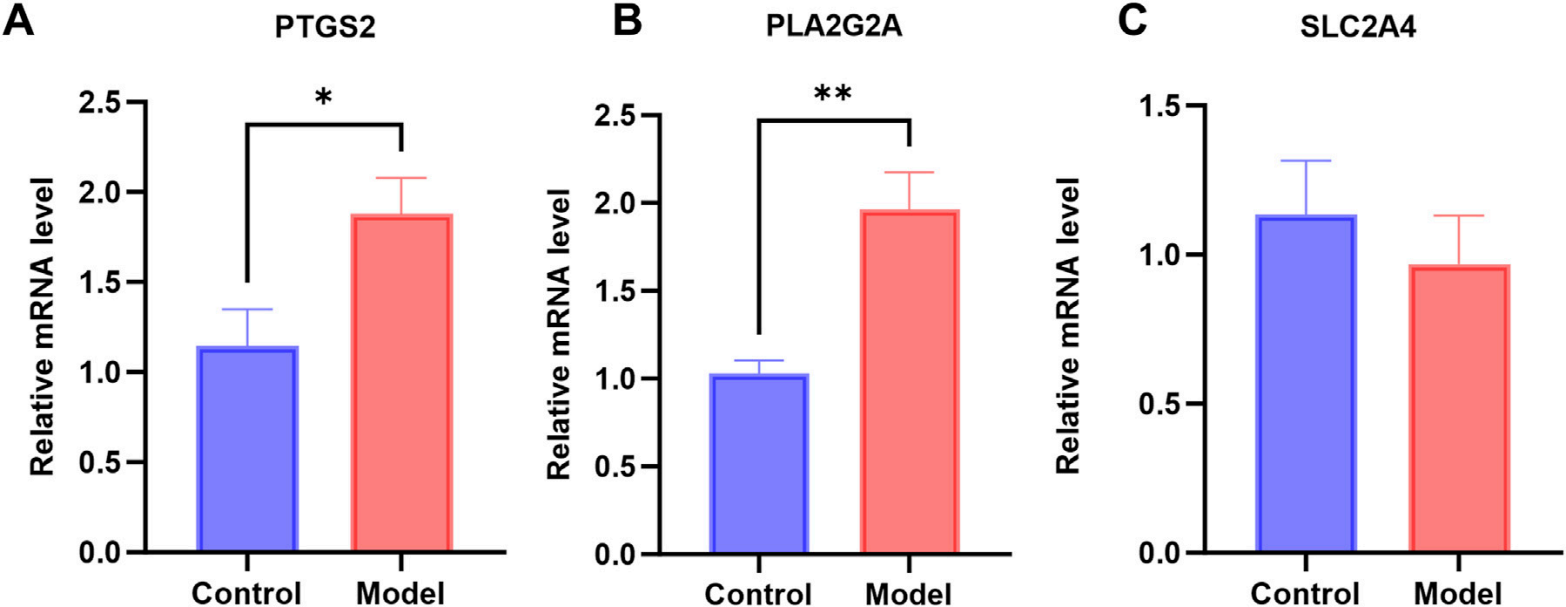
Levels of gene expression in CSU mouse models: **(A–C)** mRNA expression levels of *PTGS2, PLA2G2A*, and *SLC2A4* in the CSU mouse model (*P < 0.05).

To validate the expression of the *PTGS2*, *PLA2G2A*, and *SLC2A4* genes, we established a mouse model of CSU-like and examined the expression of these genes in skin tissues at the mRNA level. As can be seen from Figures 7A–C, the mRNA expression levels of *PTGS2* and *PLA2G2A* genes in the CSU-like model group were significantly increased, with significant differences compared with the control group (*P* < 0.05), which was consistent with the results of bioinformatics analysis. Thus, *PTGS2* and *PLA2G2A* may serve as promising therapeutic targets for CSU.

### 3.3 Non-targeted metabolomics analysis

**FIGURE 8:**
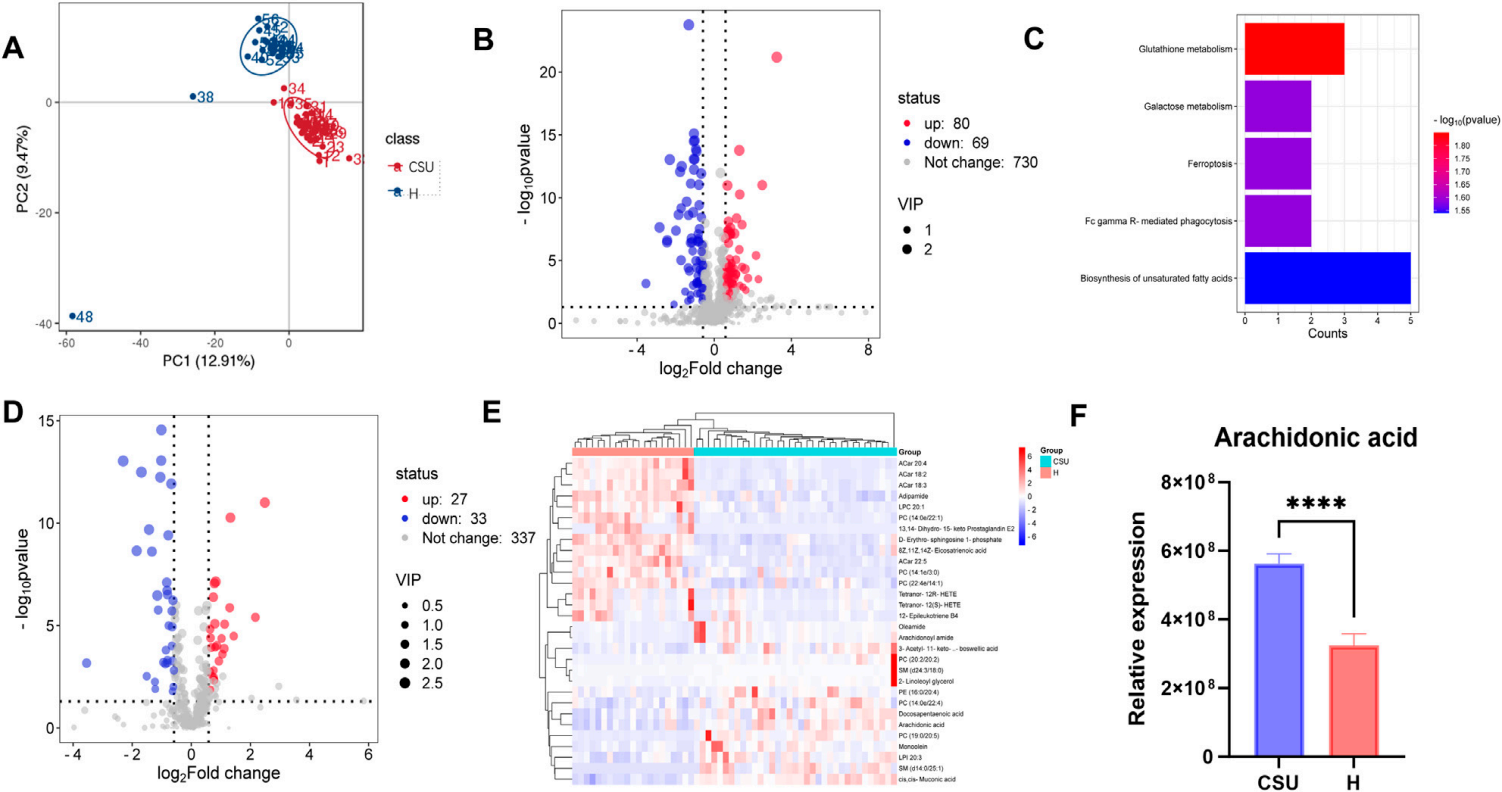
Analysis of non-target metabolomics. **(A)** PCA analysis of overall metabolites between the CSU and healthy (H) control groups. **(B)** Volcano plot of overall differential metabolites. **(C)** Kyoto Encyclopedia of Genes and Genomes (KEGG) enrichment analysis of overall differential metabolites. **(D)** Volcano plot of differential lipid-related metabolites. **(E)** Heatmap of the top 30 differential lipid-related metabolites, Blue represents significantly downregulated lipid-related metabolites, and red represents significantly upregulated lipid-related metabolites. **(F)** Arachidonic acid differential comparison (****P < 0.0001).

To examine the overall metabolic profile of CSU patients, we collected serum samples from 35 CSU patients and 21 healthy individuals for non-targeted metabolomics analysis. Principal component analysis (PCA), presented in Figure 8A, revealed distinct metabolic differences between the two groups. The volcano plot of differential metabolites (Figure 8B) identified 80 upregulated and 69 downregulated metabolites, with most of the changes related to lipid metabolism. KEGG pathway enrichment analysis (Figure 8C) indicated that unsaturated fatty acid metabolism was the most enriched pathway. These findings highlight a strong connection between lipid metabolism and the pathogenesis of CSU.

To gain deeper insights into the lipid metabolic profile of CSU patients, we compared the levels of lipid-related metabolites between the two groups. As shown in the volcano plot (Figure 8D), 27 lipid-related metabolites were upregulated, while 33 were downregulated. The top 30 differential metabolites are presented in Figure 8E. Notably, sphingomyelins (SM d14:0/25:1 and SM d24:3/18:0), phosphatidylcholines (PC 20:2/20:2 and PC 19:0/20:5), and arachidonoyl amide were upregulated, whereas 13,14-Dihydro-15-keto Prostaglandin E2 and Acylcarnitines (ACar 18:2, ACar 22:5, and ACar 20:4) were downregulated. Additionally, arachidonic acid was found to be significantly elevated in the CSU group (Figure 8F). Given that our bioinformatics analysis identified *PTGS2* and *PLA2G2A* as key genes involved in arachidonic acid metabolism, these findings suggest that arachidonic acid could serve as a potential biomarker for CSU.

## 4 Discussion

Recent studies have highlighted that abnormal lipid metabolism is associated with a variety of diseases. In this study, we delve into the link between lipid metabolism and CSU. Through the integration of bioinformatics analysis and untargeted metabolomics, we identified critical therapeutic targets and potential biomarkers for CSU. Our findings reveal that two LMRGs, *PLA2G2A* and *PTGS2*, are significantly upregulated in CSU patients compared to healthy controls. These results were further validated in a CSU-like mouse model, confirming their alignment with the bioinformatics predictions. Additionally, Gene Set Enrichment Analysis (GSEA) identified both genes as participants in the AA metabolism pathway. Our metabolomics analysis also detected elevated levels of arachidonic acid in the CSU group, reinforcing its potential role as a biomarker for CSU.

CSU is a distressing inflammatory skin disorder marked by immune cell infiltration (Maurer et al., 2024). Mast cell activation and degranulation play a key role in the development of CSU, contributing to the recurring formation of wheals and vascular edema (Kolkhir et al., 2022a). Skin mast cells facilitate the recruitment of neutrophils by modulating processes such as enhancing vascular permeability and releasing chemokines (Kulthanan et al., 2023). Neutrophils, the most abundant innate immune cells, are implicated in chronic inflammation and autoimmune disorders (Chiang et al., 2019). Although the exact relationship between neutrophils and CSU remains unclear, some studies have reported increased neutrophil infiltration in the skin lesions of CSU patients (Martins et al., 2018). Our bioinformatics analysis supports these findings, predicting elevated levels of activated mast cells and neutrophils in CSU-affected skin, along with a positive correlation between these two cell types. Additionally, we observed that the hub genes *PLA2G2A* and *PTGS2* are positively associated with both activated mast cells and neutrophils.

Previous studies have utilized bioinformatic approaches to investigate potential therapeutic targets for CSU. For instance, research by Wenxing Su et al. identified and suggested that *TNF, NF-κB, JAK-STAT, IL-6, TLR-4, ICAM-1*, and *PTGS-2* may represent novel candidate targets for CSU treatment (Su et al., 2023). Similarly, a study by Yihui Chen et al. discovered 7 upregulated hub genes (*PTGS2, CCL2, IL1B, CXCL1, IL6, VCAM1, ICAM1*) and 1 downregulated hub gene (*PECAM1*) (Chen et al., 2024), these studies employed datasets consistent with those used in our current research. However, due to methodological differences, the results obtained exhibit variations. Notably, these prior investigations did not specifically incorporate or validate the role of lipid metabolism genes in CSU pathogenesis. Interestingly, our findings converge with the aforementioned studies on one key gene: *PTGS2*, which was also identified as upregulated in our analysis. This convergence provides validation for the reliability of our results. Furthermore, our study revealed the upregulation of an additional gene, *PLA2G2A*. Significantly, both *PTGS2* and *PLA2G2A* are key genes involved in arachidonic acid metabolism.

*PLA2G2A*, also known as *sPLA2IIA*, is an enzyme responsible for hydrolyzing the sn-2 acyl ester bonds of glycerophospholipids in lipoproteins and cell membranes, producing non-esterified fatty acids and lysolipids (LPL) (Khan and Ilies, 2023; Kuzmina et al., 2022). It plays a critical role in a variety of processes within cells, including phospholipid metabolism, host defense, and signaling (Murakami et al., 2017). *sPLA2IIA* can also hydrolyze phospholipids in extracellular microvesicles, generating lipid mediators such as LPL and AA (Boudreau et al., 2014; Seeds and Bass, 1999). AA is essential for maintaining cell membrane fluidity, selective permeability, and flexibility, supporting the functionality of cells, especially within the nervous, immune, and vascular systems (Kursun et al., 2022). *PTGS2*, also known as cyclooxygenase-2 (*COX-2*), converts AA into pro-inflammatory lipid mediators, such as prostaglandins. These mediators contribute to inflammation by inducing fever, increasing vascular permeability and dilation, and promoting the release of inflammatory factors (Innes and Calder, 2018; Simon, 1999). Among these, Prostaglandins E2 (PGE2) and Prostaglandin D2 (PGD2) play an important role, PGE2 mediates mast cell recruitment through EP3 receptors (Weller et al., 2007). While synchronously orchestrating T cell polarization via EP2/EP4 dependent pathways potentiating Th1/Th17 differentiation and Th22-derived IL-22 secretion to establish a pro-inflammatory milieu conducive to autoimmune pathogenesis (Tsuge et al., 2019). PGD2 is the predominant prostanoid secreted by activated mast cells and has long been associated with allergic diseases. (Hirai, 2004). In turn, mast cell activation induces further *COX-2* expression and promotes PGE2 production (He et al., 2001; Hennino et al., 2006).

Mast cell activation and degranulation are primary drivers of CSU pathogenesis. In summary, *PLA2G2A* and *PTGS2* are crucial components of arachidonic acid metabolism, a finding supported by our GSEA, which confirmed their joint involvement in this pathway. Furthermore, previous research has demonstrated a strong association between elevated *PTGS2* expression and the onset of urticaria (Chen et al., 2024; Su et al., 2023), consistent with our observations. Validation of these two genes in the CSU mouse model further aligns with the results of our bioinformatics analysis.

Previous serum metabolomic analyses have demonstrated alterations in serum levels of docosahexaenoic acid (DHA), arachidonic acid, glutamate, and succinate in patients with CSU (Wang et al., 2020). Furthermore, investigations into plasma lipid metabolism in chronic urticaria patients revealed significant changes in plasma glycerophospholipids, characterized by marked upregulation of phosphatidylserine (PS), phosphatidylethanolamine (PE), and phosphatidylglycerol (PG), alongside significant downregulation of phosphatidylcholine (PC) (Li et al., 2022). Collectively, these findings indicate a significant association between CSU and dysregulation of lipid metabolism. We conducted untargeted metabolomic analysis on serum samples from CSU patients and healthy individuals. The results showed that lipid metabolites comprised the majority of differentially expressed metabolites, with polyunsaturated fatty acid (PUFA) metabolism identified as the most enriched KEGG pathway. PUFAs play a vital role in cell function and development across both eukaryotes and prokaryotes (Czumaj and Śledziński, 2020). AA, a key PUFA, has both endogenous and exogenous sources. Endogenously, AA is derived from membrane phospholipids through the catalytic action of enzymes from the phospholipase A2 (PLA2) superfamily, which are activated by cellular signals such as tumor necrosis factor receptor (TNFR) and Toll-like receptor 4 (TLR4) stimulation (Dennis et al., 2011). These receptors are notably upregulated in CSU patients (Fang et al., 2022; Gao et al., 2011), suggesting that inflammatory stimuli in CSU can trigger AA release. Previous studies have demonstrated that applying solvent-dissolved AA to mouse skin induces edema, vascular permeability, and immune cell infiltration, accompanied by elevated levels of PGE2, thromboxane B2 (TxB2), and leukotriene B4 (LTB4) (Rao et al., 1993). Furthermore, increased free AA levels have been reported in inflamed psoriatic skin tissues (Brash, 2001). Consistent with these findings, our analysis also revealed significantly higher AA levels in CSU patients compared to healthy controls. Given that *PLA2G2A* and *PTGS2* are central to AA metabolism, we propose that AA may be metabolized by these enzymes into downstream inflammatory lipid mediators, such as PGD2 and PGE2*.* Relevant literature indicates several clinical associations, PGD2 levels are elevated in local venous blood from patients with cold urticaria, and its production is associated with human mast cell degranulation (Heavey et al., 1986). In allergic inflammation, activated mast cells synthesize PGD2, which exerts bronchoconstrictive and vasodilatory effects and attracts neutrophils, inducing their aggregation (Rasković et al., 1998)*.* Furthermore, studies have shown that PGE2 levels are significantly higher in patients with CSU compared to healthy individuals (Rasković et al., 1998). These mediators could lead to vasodilation, fluid exudation, enhanced vascular permeability, and the accumulation of inflammatory cells, driving the development of CSU.

Although our study has made some progress, certain limitations remain. First, the limited sample size available in public databases may impact the reliability of our findings. Second, inherent methodological challenges in hub gene identification persist: For instance, most algorithms rely on arbitrarily defined connectivity thresholds (e.g., selecting top 10 or 20 genes by degree) lacking unified biological criteria (Bo et al., 2022; Xie et al., 2022), with no consensus on optimal cutoffs. This arbitrariness not only causes substantial variability across studies, hindering comparability and reproducibility, but may also overlook functionally important genes with connectivity slightly below thresholds. Furthermore, computational models and network construction methods exhibit significant heterogeneity: Some studies employ machine learning-based feature selection (e.g., LASSO regression) to screen feature genes predictive of disease states (Liu et al., 2021). Others identify topologically central protein-coding genes using PPI network databases (Xie et al., 2022). WGCNA focuses on constructing co-expression modules to identify intramodular hub genes (Pan et al., 2016). These methodological divergences frequently yield discordant results. Third, the precise roles of these genes and metabolites in the pathogenesis of CSU remain unclear. Critically, these genes and lipids may merely serve as markers of mast cell activity, potentially representing consequences of the disease rather than causative factors. Thus, further experiments are required to analyze their mechanisms of action and establish causal relationships. We will conduct comprehensive functional validation studies—including gene knockout and overexpression assays—to definitively establish their biological functions, pathophysiological causal roles, and molecular regulatory mechanisms in disease pathogenesis.

## 5 Conclusion

In conclusion, this study identified and analyzed two LMRGs, *PTGS2* and *PLA2G2A*, which may be hub genes for the onset of CSU. Furthermore, we propose arachidonic acid as a promising biomarker for the condition. Therefore, our findings provide valuable insights and offer new directions for future diagnostic and therapeutic strategies for CSU.

## Data Availability

The original contributions presented in the study are included in the article/Supplementary Material, further inquiries can be directed to the corresponding authors.
