# Supplementary material for: Bioinformatics analysis combined with untargeted metabolomics reveals lipid metabolism-related genes and their biological markers in chronic spontaneous urticaria
Source: Front Genet. 2025 Aug 18;16:1550205. doi: 10.3389/fgene.2025.1550205 (PMC12399643; doi:10.3389/fgene.2025.1550205)
Supplement: Supplementary file 1 [file Supplementaryfile1.docx]

*
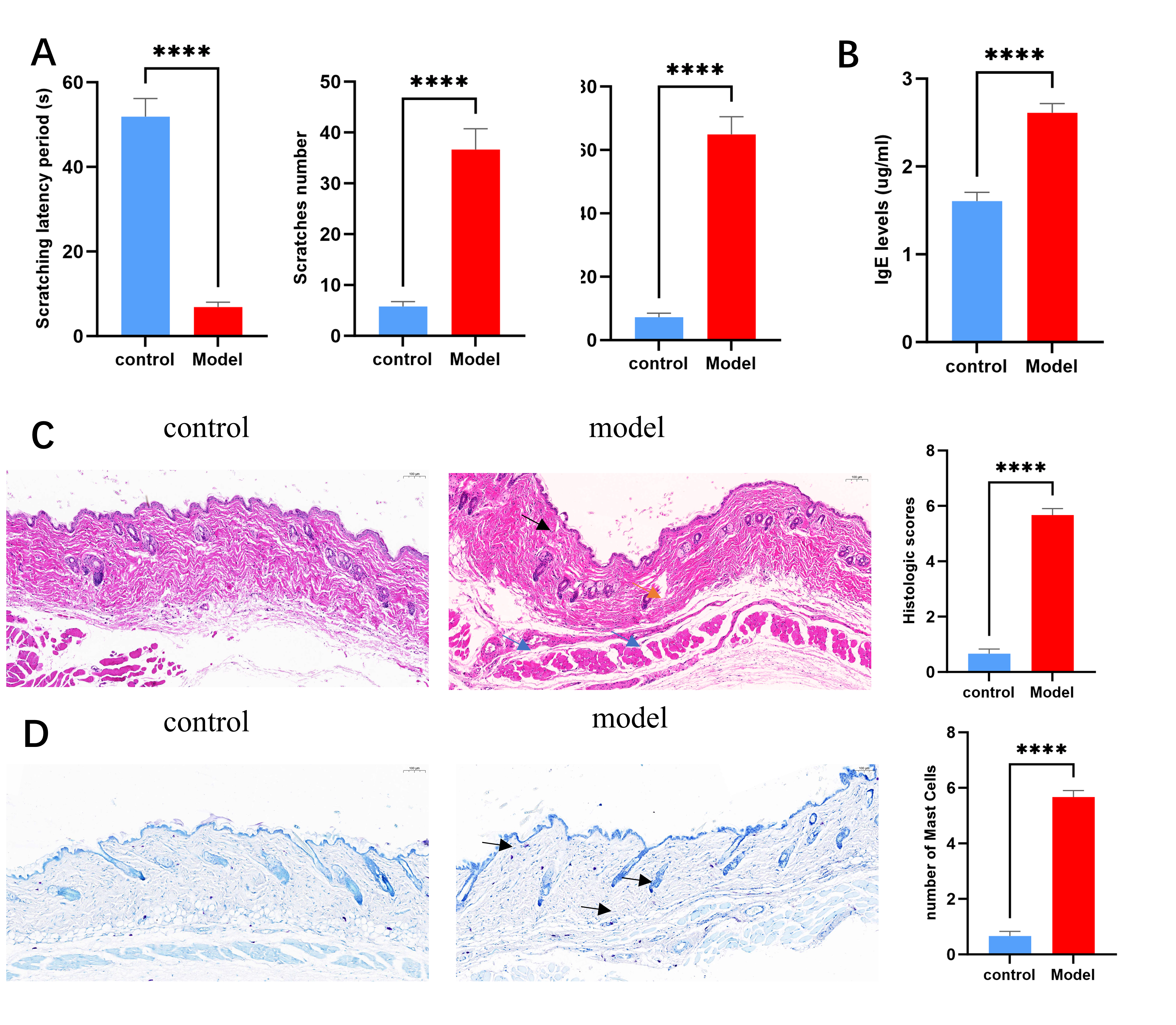
*

Fig. S1 Behavioral, Serum IgE, and Histopathological Manifestations in the OVA-Induced Mouse Model. A Behavioral changes in the OVA-induced mouse model. B Serum IgE levels in the OVA-induced mouse model. **C**  Histopathological alterations in the OVA-induced mouse model (Black arrows: Inflammatory cells; Yellow arrows: Tissue edema and widened interstitium; Blue arrows: Dilated and congested capillaries). **D** Mast cell changes in the OVA-induced mouse model (Black arrows indicate mast cells) (*****P* < 0.0001)
