# Supplementary material for: Bioinformatics analysis combined with untargeted metabolomics reveals lipid metabolism-related genes and their biological markers in chronic spontaneous urticaria
Source: Front Genet. 2025 Aug 18;16:1550205. doi: 10.3389/fgene.2025.1550205 (PMC12399643; doi:10.3389/fgene.2025.1550205)
Supplement: Supplementary file 2 [file Table1.docx]

Additional file 1. Table S1. Real-time quantitative PCR primer sequences.

| Symbol | Primer Sequence |
| --- | --- |
| *PTGS2* | forward: 5'- TTCAACACACTCTATCACTGGC -3' |
|  | reverse: 5'- AGAAGCGTTTGCGGTACTCAT -3' |
| *PLA2G2A* | forward: 5'- TCTGCATTTGTCACCCAAGAACTCT -3' |
|  | reverse: 5'- ATTCAGCACTGGGTGGAAGGTTT -3' |
| *SLC2A4* | forward: 5'- GTGACTGGAACACTGGTCCTA -3' |
|  | reverse: 5'- CCAGCCACGTTGCATTGTAG -3' |
