# Supplementary material for: Bioinformatics analysis combined with untargeted metabolomics reveals lipid metabolism-related genes and their biological markers in chronic spontaneous urticaria
Source: Front Genet. 2025 Aug 18;16:1550205. doi: 10.3389/fgene.2025.1550205 (PMC12399643; doi:10.3389/fgene.2025.1550205)
Supplement: Supplementary file 3 [file Table2.docx]

**Table S2 Demographic and clinical characteristics of participants.**

| Features | CSU patients（n=35） | H（n=21） | *p*-value |
| --- | --- | --- | --- |
| Age (years), mean ± SD | 37.5±13.9 | 39.9±9.4 | 0.40 |
| Sex (female) *,n（%）* | 20(57.1) | 13(61.9） | 0.79 |
| BMI (kg/m2), mean ± SD | 21.36±1.7 | 20.8±1.6 | 0.10 |
| Time since diagnosis of CSU (months), median (IQR) | 21(3-24) | - | - |
| Age of onset CSU (years), mean ± SD | 34.6±15.3 | - | - |
| Resistance to antihistamines *,n（%）* | 4(11.4) | - | - |
| Gastrointestinal symptoms during the onset of urticaria *,n（%）* | 6(17.1) | - | - |
| History of food allergy*, n（%）* | 10(28.6) | - | - |
| History of drug allergy*, n（%）* | 4(11.4) | - | - |
| Family history of urticaria*, n（%）* | 5(14.3) | - | - |

CSU, chronic spontaneous urticaria; H, healthy controls; BMI, body mass index; IQR, interquartile range.
